# Low-Cost and Readily Available Tissue Carriers for the Boston Keratoprosthesis: A Review of Possibilities

**DOI:** 10.1155/2013/686587

**Published:** 2013-11-24

**Authors:** Andrea Cruzat, Allyson Tauber, Anita Shukla, Eleftherios I. Paschalis, Roberto Pineda, Claes H. Dohlman

**Affiliations:** Cornea & Refractive Surgery Service, Massachusetts Eye & Ear Infirmary, Department of Ophthalmology, Harvard Medical School, 243 Charles Street, Boston, MA 02114, USA

## Abstract

The Boston keratoprosthesis (B-KPro), currently the most commonly used artificial cornea worldwide, can provide rapid visual rehabilitation for eyes with severe corneal opacities not suitable for standard corneal transplantation. However, the B-KPro presently needs a corneal graft as a tissue carrier. Although corneal allograft tissue is readily available in the United States and other developed countries with established eye banks, the worldwide need vastly exceeds supply. Therefore, a simple, safe, and inexpensive alternative to corneal allografts is desirable for the developing world. We are currently exploring reasonable alternative options such as corneal autografts, xenografts, noncorneal autologous tissues, and laboratory-made tissue constructs, as well as modifications to corneal allografts, such as deep-freezing, glycerol-dehydration, gamma irradiation, and cross-linking. These alternative tissue carriers for the B-KPro are discussed with special regard to safety, practicality, and cost for the developing world.

## 1. Introduction

**Figure 1:**
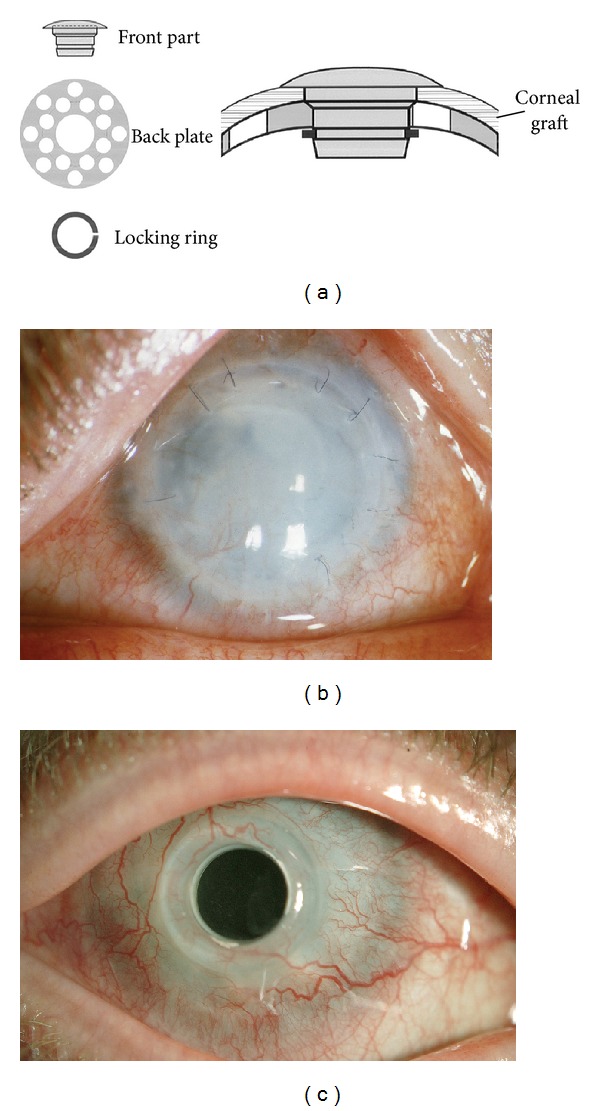
(a) Assembly of the Boston keratoprosthesis. (b) Patient with failed corneal graft due to candida infection (c) 13 years postoperatively vision 20/30.

The Boston keratoprosthesis (B-KPro) is an artificial cornea that offers a viable solution for corneal transplant candidates who are at high risk for graft failures such as those with a prior history of graft rejection, dry eyes, and severe neurotrophic and autoimmune diseases. It provides a clear visual axis without astigmatism and rapid visual recovery postoperatively. It is the most widely used corneal prosthesis in the United States and in the rest of the world [1]. The B-KPro has a collar-button design with a front plate, stem, and back plate of poly[methyl methacrylate] (PMMA) or titanium [2]. The device is implanted into a corneal graft and then sutured into the patient's cornea as in standard penetrating keratoplasty (Figure 1). The Boston type I procedure is favored in eyes with adequate tear secretion, whereas the type II B-KPro (with an added anterior nub) is reserved for near-hopeless cases with severe destruction of the ocular surface, such as end-stage dry eye conditions and cicatricial diseases [3]. 

From a global perspective, the need for human corneas far exceeds supply. Although corneal tissue is readily available in many regions of the developed world with established eye bank systems, this is not the case for other populations. In many developing countries, cultural and religious concerns limit organ donations [4, 5]. Furthermore, healthcare and financial restrictions can be a major barrier: for example, a donor cornea from an eye bank in the United States can cost about $3,000 due to the need for microbial testing, administration, and transport alone [6]. Thus, the lack of human donor tissue at a reasonable cost is one of the largest barriers to reducing blindness through either standard PK or KPro implantation [7]. A long-term, safe, and inexpensive alternative is clearly needed for the developing world. 

Why is it necessary to use a corneal graft as a vehicle for the B-KPro? After all, several other artificial corneas, proposed from other centers throughout history, have been designed to be implanted directly into the recipient's diseased cornea without using a graft as a carrier. The answer lies in our experience that a double-plated (collar-button) keratoprosthesis design has advantages over designs where the optical stem is anchored by a horizontal haptic (plastic, metal, tooth, etc.) placed *within* or *in front* of the patient's cornea. According to our results, positioning of the B-KPro's back plate entirely *behind* the corneal tissue, with its intact Descemet's membrane, provides better long-term retention than other arrangements. Accepting this principle of positioning, we have in the past tried to work out practical techniques for direct insertion into the patient's cornea without using a graft but without success. Such approaches have been too traumatic at surgery or resulted in early tissue melt. Therefore, we have used a corneal graft as a vehicle for the device since the mid-1960s, with improved retention.

We are therefore exploring alternatives to fresh corneal tissue for use as a keratoprosthesis carrier. This includes physical alterations to increase storage time, such as deep-freezing, glycerol-dehydration, gamma irradiation, or cross-linking of tissue. Other substitutes may include corneal autografts, xenografts, skin, cartilage, and tissue constructs. This review identifies alternative tissue carriers suitable for the developing world in terms of safety, practicality, and cost effectiveness. 

## 2. Carriers for the Boston Keratoprosthesis

In the developed world with high levels of health expenditures and established eye banks, standard fresh corneal allografts (preserved from 1 to 14 days) are preferred. Additionally, allografts allow assembly with the B-KPro on a side table before opening the patient's eye. This minimizes the time the eye is open and risk of vitreous protrusion or choroidal hemorrhage. But, in the developing world, issues with cost, logistics, and administration of the donor graft are very substantial. 

One alternative is to modify the donor tissue in order to lengthen storage time prior to use. It is known that an allograft can be stored *deep-frozen* (thus nonviable) and still be usable as a lamellar graft [8]. As a carrier for the B-KPro, there was no demonstrable difference in clinical outcomes between fresh and frozen donor materials in a recent large series [9]. Deep-freezing destroys all corneal cells and should theoretically be less antigenic. Storing human allogenic corneas in a hospital freezer is practical for the B-KPro surgery locally, but transport remains a problem. 

A second alternative may be to use *dehydrated* rather than frozen corneal tissue, where shipment in a small vial is easy and practical see Table 1. Dehydration is performed simply by replacing corneal water with glycerol and has long been a standard technique in biology [10]. Glycerol-preservation of donor corneal tissue has for many decades been used in lamellar keratoplasty with satisfactory outcomes [11]. This method extends the time that a cornea can be stored and can thereby allow the use of tissue that would otherwise be discarded on the basis of prolonged death-to-preservation time. In 2008, the Eye Bank Association of America estimated that the United States harvested over 92,000 corneas, including 30,000 that were deemed unsuitable for optical grafting. Of these, approximately 25% could have been preserved with glycerol and eventually used, increasing the cornea donor pool by 7,000–8,000 tissues annually [7]. Our experience with glycerol-stored corneas in B-KPro surgery has been favorable but the corneas need to be rehydrated in saline for 30–60 minutes before use; otherwise their leathery consistence makes suturing difficult. Corneas dehydrated by freeze-drying, rather than by glycerol, would be expected to have similar qualities, but preparation and shipment would be more cumbersome.

Corneal allografts can be modified in other ways. After B-KPro implantation, graft necrosis, melt, and subsequent leak can occur postoperatively, particularly in autoimmune diseases, leading to calamities like retinal detachment or infection. Pretreatment aimed at increasing resistance to enzymatic digestion might help reduce the incidence. Cross-linking corneas with the help of riboflavin plus UV-A light has been shown to reduce corneal susceptibility to digestion with collagenases [12]. Whether such modified corneal grafts show increased long-term stability is presently the subject of a clinical study. 

*Gamma irradiated* human corneas, such as Visiongraft Sterile Cornea, available from Tissue Banks International, have recently been used in place of fresh donor corneas for lamellar patch grafts [13]. They have also been used successfully as carriers for the B-KPro [14]. In our experience, these grafts are thinner than a fresh graft but are still functional. In addition, these corneas can be easily transported in a vial at room temperature. However, as long as testing for residual viruses is required, the cost remains high, and it is questionable whether gamma irradiated corneas have any advantage over glycerol-dehydrated tissue.

For countries with limited resources and no eye bank administration, the use of the patient's own cornea (*autograft*) has obvious advantages. The graft and host trephining diameters will, by necessity, be the same. The B-KPro assembly will have to take place on a side table with the patient's eye open, which should take a maximum of five minutes. In spite of such relatively minor disadvantages, the benefits of low cost and easy logistics are often overriding, and this approach is in use in several countries. A recent pilot keratoprosthesis program was initiated in Ethiopia and Sudan that successfully used patients' own corneas as carriers for the B-KPro [15]. While these results are promising, widespread adoption is limited by the health of the presurgical cornea which must not have any stromal melt, perforation, or infection. Often, the cornea is too damaged and thin to be used. Complications are apt to occur if corneas are unable to swell and fill the space between the B-KPro plates, compounded by a heightened sensitivity to necrosis and melt in corneas severely damaged from disease. Therefore, we recommend the patient's own cornea as a carrier only when it is still capable of swelling and is not too scarred, inflamed, or thin [15]. 

A thin and scarred cornea can be used as an autograft, however, if covered by a large conjunctival flap [16]. Such a flap should be mobilized vertically, from the temporal sclera, with intact bases above and below. It should be moderately thick and wide enough to fit loosely over the entire cornea. It is important that all corneal epithelium be removed before the flap is sutured down (with 10-0 nylon) to the limbus, so that a pure connective tissue interface is created. Leaving patches of epithelium behind would later result in epithelial cysts. A small central opening can be made in the flap at the end of surgery and will result in gradual retraction of the conjunctival edge to the edge of the B-KPro front plate. Such a flap is an effective way of covering the carrier cornea with a much needed barrier of conjunctival epithelium and subepithelial blood vessels, ideally preventing any subsequent tissue melt. A thick flap has the added benefit that it can make soft contact lens wear unnecessary. The downside of such a vertical conjunctival flap is that it makes the procedure more complex by adding surgery time. Also, there are situations where the conjunctiva is too thin and scarred down to be mobilized. 

A hypothetical carrier possibility would be to use an *extracorneal autologous tissue*, such as skin. Thin, hairless dermis can be harvested from behind the ears or elsewhere. Likely a basement membrane (e.g., amnion) between the graft and the back plate will be needed in order to block cells and large proteins from entering the eye through the plate holes. Such studies are presently being conducted in animals. Also, various forms of cartilage in the body are being explored as carrier graft substitutes shaped into suitable diameter and thickness as either autografts, allografts, or xenografts [17]. In prior studies, cartilage from sternum or ears has been implanted in animal corneas [18, 19]. 

It is important to acknowledge that not only availability and safety but also low cost is a requirement for B-KPro carrier tissue in the developing world. For many patients, neither autografts nor allografts are feasible, forcing us to look beyond those possibilities. One such possibility is xenografts (tissue from another species). Gamma irradiated porcine and bovine heart valves, pericardium, and so forth have been successfully used in humans for years. If corneas from such animals, ubiquitous in barnyards all over the world, could be tolerated in human eyes, the search for a suitable carrier material would be over. The biological questions regarding xenografts would be transplantation immunity and sterility. Gamma irradiation reduces antigenicity and sterilizes the tissue, making xenografts a real possibility for humans.

Xenotransplantation from pigs is appealing with respect to the cornea, as there are many similarities to humans with regards to refractive power, size, and tensile strength [20]. Recent genetic manipulation of pigs has led to the prospect that the remaining immunological barriers will be overcome [21]. Though there is less risk of rejection from genetically manipulated pigs, the cost is still high and availability is complex. The question of sterility is also of high importance and whether gamma irradiation (25–35 KGy) can kill not only bacteria but also all viruses that can pose a potential threat to the new host. If the surface of such a xenograft would still be vulnerable to necrosis and melt, a conjunctival flap as described above, should be protective. These hypotheses are presently being tested by our group in animal experiments [22].

Finally, laboratory-synthesized tissue constructs may be an alternative carrier tissue. Sheets of collagen fibrils, moderately cross-linked, have been successfully used as lamellar grafts in humans with keratoconus [23, 24]. Recent studies on the safety and functionality of a biosynthetic cornea found that it supported the ingrowth of endogenous cells and nerves, as well as functions such as touch sensation, development of tear film, and normal eye pressure [23, 25]. Theoretically, the use of such constructs would be very attractive as costly microbiological testing would not be necessary, manufacturing costs would likely decline over time once mass produced, and shipping would be easy. The present difficulty seems to be making the constructs biocompatible, thick, and cohesive enough for suturing into a recipient cornea.

In addition to expanding available options for B-KPro carrier tissue, efforts are under way to make the B-KPro device more readily available for the developing world and the postoperative care less expensive. New designs of the B-KPro that are more cost-effective are under current development. Low-cost bandage contact lenses of variable diameters are recommended to be used with the B-KPro. Although antibiotic prophylaxis is needed for life in a patient with a B-KPro, simple and affordable antibiotic combinations are suggested such as Polymyxin B + Trimethoprim sulfate daily to diminish the financial burden of this approach.

## 3. Conclusion

Of the estimated worldwide 4–10 million people with corneal blindness needing surgical rehabilitation, at least 80% live in the developing world [5, 26]. For this underserved population, the B-KPro is expected to play an increasing role. The present surgical technique of implantation requires corneal tissue as a carrier for the device—however, the high cost and lack of availability constitute a severe hurdle in many countries. There is therefore an urgent need to find alternative carrier tissues that must be inexpensive, readily available, and safe. In this review, we have presented a number of alternative options and their advantages and disadvantages are discussed.

## Figures and Tables

**Table 1:** Possible tissue carriers for the Boston keratoprosthesis and predicted qualities.

Donor tissue	Potential availability	Potential cost	Preparation of tissue	Ease of shipment, storage	Complexity of surgery	Risk of infection	Expected antigenicity	Likelihood of necrosis
Autograft	Uneven	None	No issue	No issue	Standard	Low	None	Low
Autograft plus conjunctival flap	Better	None	No issue	No issue	Complex	Very low	None	Very low
Skin autograft	Good	None	Skin excision	No issue	Complex	Low	None	?
Allograft, fresh	Uneven	High	Standard	Complex	Standard	Low	Low	Low
Allograft, frozen	Uneven	High	Deep-freeze	Complex	Standard	Low	Very low	Low
Allograft, dehydrated	Good	High	Dehydration (in glycerol or freeze-dried)	Good	Time to rehydrate	Low	Very low	Low
Allograft, X-linked	Good	High	X-linking	Complex	Standard	Low	Low	Low
Allograft, *γ*-radiated	Good	High?	*γ*-radiation	Good	Standard	Low	Very low	Low
Xenograft, *γ*-radiated (tested for viruses)	Good	High	*γ*-radiation	Good	Standard	Low	?	?
Xenograft, *γ*-radiated (untested for viruses)	Good	Low	*γ*-radiation	Good	standard	?	?	?
Xenograft *γ*-radiated (untested) (plus conjunctival flap)	Good	Low	*γ*-radiation	Good	Complex	?	?	Low
Collagen constructs	Good	Low	Manufacturing	Good	Complex?	Low	Very low	?
